# Posterior urethral valves: Morphological normalization of posterior urethra after fulguration is a significant factor in prognosis

**DOI:** 10.4103/0971-9261.71744

**Published:** 2010

**Authors:** Prema Menon, K. L. N. Rao, S. Vijaymahantesh, R. P. Kanojia, R. Samujh, Y. K. Batra, K. S. Sodhi, A. K. Saxena, A. Bhattacharya, B. R. Mittal

**Affiliations:** Department of Pediatric Surgery, Advanced Pediatric Center, Post Graduate Institute of Medical Education and Research, Chandigarh, India; 1 Department of Anesthesiology, Advanced Pediatric Center, Post Graduate Institute of Medical Education and Research, Chandigarh, India; 2 Department of Radio-Diagnosis, Advanced Pediatric Center, Post Graduate Institute of Medical Education and Research, Chandigarh, India; 3 Department of Nuclear Medicine, Advanced Pediatric Center, Post Graduate Institute of Medical Education and Research, Chandigarh, India

**Keywords:** Cystourethrogram, hydronephrosis, posterior urethral valves, vesicoureteral reflux, voiding dysfunction, valve bladder

## Abstract

**Aim:**

To assess the changes in urethral morphology 3 months post fulguration of posterior urethral valves (PUVs) on micturating cystourethrogram (MCUG) and correlate these changes with the overall clinical status of the patient.

**Materials and Methods:**

A total of 217 children, managed for PUVs during a period of 6 years in a single surgical unit were prospectively studied. The ratio of the diameters of the prostatic and bulbar urethras (PU/BU) was calculated on the pre- and post-fulguration MCUG films. They were categorized into three groups based on the degree of normalization of posterior urethra (post-fulguration PU/BU ratio).

**Results:**

Group A: Of the 133 patients, 131 had normal urinary stream and 4 (3%) had nocturnal enuresis. Vesicoureteral reflux (VUR), initially seen in 83 units (31% units), regressed completely at a mean duration of 6 months in 41 units (49%). Of the 152 non-VUR, hydroureteronephrosis (HUN) units, 11 were poorly functioning kidneys. Persistent slow but unobstructed drainage was seen in 23 units (16%) over a period of 1.5–5 years (mean 2.5 years). Group B: All the 11 patients had a normal stream. Four (36.4%) had daytime frequency for a mean duration of 1 year and one (9%) had nocturnal enuresis for 1 year. Grade IV–V VUR was seen in five patients (three bilateral), which regressed completely by 3 months in five units (62.5%). In the non-VUR, HUN patients, slow (but unobstructed) drainage was persistent in two units (14%) at 3 years. Group C: Of the 16 patients, only 5 (31.3%) were asymptomatic. Six patients (nine units) had persistent VUR for 6 months to 3 years. Of the 20 units with HUN, 17 (85%) were persistent at 1–4 years (mean 2 years). Eight patients (50%) required a second fulguration while 3 (18.7%) required urethral dilatation for stricture following which all parameters improved.

**Conclusions:**

Adequacy of fulguration should be assessed by a properly performed MCUG. A postop PU/BU ratio >3 SD (1.92) should alert to an incomplete fulguration or stricture. Patients within normal range ratio have faster recovery of slow draining units, reflux and less voiding dysfunction. There is a strong correlation between incomplete fulguration and persistent slow draining units, uremia, voiding dysfunction and urinary tract infections.

## INTRODUCTION

Posterior urethral valves (PUVs) have a variable effect on the anatomy and physiology of the bladder and the upper urinary tract. Many variables play their role in the ultimate prognosis. Fulguration is well accepted as the primary mode of management of PUVs. However, very few authors have studied the changes in the anatomical configuration of the posterior urethra (PU) after fulguration. Moreover, correlation of these changes with the postoperative general condition, voiding status, and improvement in function and drainage of the upper urinary tract has hardly been discussed in the literature. We carried out this study to assess the changes in the urethral morphology 3 months post fulguration and correlate these changes with the clinical status of the patient and to identify guidelines for a second fulguration if required based on the dimensions of the anterior and posterior urethera.

## MATERIALS AND METHODS

This was a 6-year prospective study from April 2004 to March 2010. All the PUV patients with preop and postop micturating cystourethrogram (MCUG) were included. A fixed protocol was followed for management. The patients who were unstable and admitted with uremia, acidosis, dehydration, etc., were first resuscitated and only then taken up for fulguration. This entailed bladder catheterization or insertion of flank drain for massive ascites or ultrasound guided percutaneous nephrostomy (PCN). If the renal function tests were normal or only slightly deranged, it was preferred to take the case for elective fulguration avoiding catheterization.

MCUG (filling in antero-posterior view and expression voiding phase in an oblique view) was done in the operation theater under general anesthesia just prior to fulguration. The patient was then placed in the lithotomy position. The urinary stream was observed on the table prior to fulguration. All the fulgurations were done by a single senior surgeon (KLNR). The valves were fulgurated at 5, 7 and 12 o’clock positions with a 9-F resectoscope (Wolf, Munich, Germany), hook electrode, cutting diathermy and usage of 1.5% glycine as the irrigating agent. Adequate fulguration was checked on table by observing the urine stream with suprapubic pressure. The bladder was catheterized for a day with a Foley catheter. MCUG was repeated 3 months after fulguration.

Group E consisted of the following patients who were also managed by us during the study period, but excluded from the analysis: (i) 7 sick neonates who died before or immediately after fulguration and (ii) 29 children with inadequate MCUGs, no follow-up or were awaiting vesicostomy/ureterostomy closure.

### Posterior urethra/bulbar urethra (PU/BU) ratio

As a control group, 50 MCUGs, taken in the oblique view, of children who had normal urinary stream and no voiding dysfunction were studied. Their age ranged from 7 days to 12 years. The maximum diameters of the posterior urethra (PU) and the bulbar urethra (BU) were measured with a standard scale in centimeters over the MCUG film. The PU/BU ratio was calculated for all the 50 patients. The mean PU/BU ratio in the controls ranged from 0.33 to 1.67, with a mean of 1.04 and standard deviation (SD) ± 0.293.

Similar ratios were calculated for all children in the study group before and after fulguration of PUV. The patients were classified into three groups depending on the post-fulguration PU/BU ratio. Group A: Less than 2 SD of normal mean, i.e., 1.04 ± 2 × 0.293 (0.454–1.626); group B: between 2 and 3 SD from normal (1.626–1.92); and group C: more than 3 SD from normal (>1.92). The bladder morphology and vesicoureteral reflux (VUR) status were also compared on the MCUG.

Group D: The patients who had previously undergone PUV fulguration elsewhere and were referred to us for further management were separately analyzed as group D.

All the patients were followed up indefinitely. Overall clinical condition, urinary stream, voiding dysfunction, urine cultures and biochemical renal function were assessed at follow-up. Postoperative diethylenetriamine pentaacetic acid (DTPA) or ethylenedicysteine (EC) and dimercaptosuccinic acid (DMSA) scans were done to check drainage patterns, function and scars at 3 monthly and then at yearly intervals.

## RESULTS

A total of 181 boys with PUVs were studied. Their age ranged from 2 days to 12 years. There were 53 (29.5%) neonates (mean 12 days), 61 (33.5%) patients between 1 month and 1 year (mean 5.5 months), 46 (25.5%) between 1 and 5 years (mean 2 years 7 months) and 21 (11.5%) between 5 and 12 years (mean 8 years 3 months). Of the 160 patients of groups A, B and C, 68 (42.5%) had associated VUR of which 47% were bilateral. A total of 16 PCNs were performed in the VUR group for pyonephrosis, poorly draining renal units or urinomas. Eleven neonates with gross ascites also had a flank drain inserted at admission to relieve respiratory distress. Thirty-six patients were excluded as mentioned in the section “Materials and Methods,” and a total of 181 patients, of whom 160 were primarily treated in our unit and 21 were referred from other centers (after first fulguration) (group D) were analyzed for changes in PU/BU ratio and the consequences on the urinary stream, voiding pattern, and function of renal units [Table 1].

**Table 1:** Comparative analysis of patient groups based on post-fulguration PU/BU ratio

	<2 SD	2–3 SD	>3 SD
Number of patients	133	11	16
Preop PU/BU ratio	1.75–32 (mean 5.82)	2.5–30 (mean 12.295)	3.5–32 (mean 10.45)
Postop PU/BU ratio	0.16–1.6 (mean 1.029)	1.67–1.86 (mean 1.734)	2–9.3 (mean 3.69)
Age	6 days–10 years (mean 10 months)	2 months–12 years (median 7 months; mean of 3.54 years)	9 days–8 years (mean 10 months)
Preop BUN (mg/dL)	11–374 (mean 67.57)	24–245 (mean 97.54)	15–240 (mean 55.4)
Preop creatinine (mg/dL)	0.4–5.6 (mean 1.37).	0.5–3.9 (mean 1.636)	0.5–3.2 (mean 1.29)
Postop BUN (mg/dL)	11–120 (mean 36.44)	11–85 (mean 30.16)	10–107 (mean 28.25)
Postop creatinine (mg/dL)	0.4–1.9 (mean 0.79)	0.4–1.8 (mean 0.74)	0.3–1.6 (mean 0.67)
Normal renal units	31	0	3
Preop VUR (units)	83	8	9
Post-fulguration VUR (units)	42	3	9
Poorly functioning units	28	2	2
Persistent VUR in functioning units	14	1	7
Preop slow draining HUN (units)	152	14	20
Poor functioning units	11	0	0
Persistent HUN (units)	23 (16%)	2 (14%)	17 (85%)
Postop poor stream, straining	2	0	11
Nocturnal enuresis	4	1	1
Daytime frequency, dribbling	7	4	8

### Group A: Postop PU/BU ratio < 2 SD, i.e., 0.454–1.626

**Figure 1:**
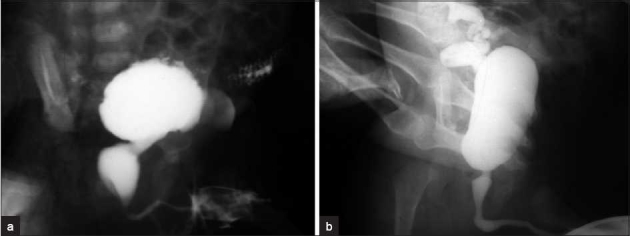
(a) Preop MCUG; (b) postop MCUG showing PU/BU ratio less than 2 SD

One hundred and thirty-three (83%) patients had a postop PU/BU ratio ranging from 0.16 to 1.6 (mean 1.029) [Figure 1a and b]. Normal urine stream was achieved in 131 children. Their preop ratio ranged from 1.75 to 32 (mean 5.82). Their age ranged from 6 days to 10 years (mean 10 months). Forty-nine patients (36.8%) were neonates, with one being a preterm at 33 weeks. Neonates and infants comprised 62% of group A. The pre-op ratio in neonates and infants was 2-32 (mean 7.89). Their post-op ratio ranged from 0.43 to 1.6 (mean 0.88). Preop blood urea nitrogen (BUN) levels ranged from 11 to 374 (mean 67.57 mg/dL) and the creatinine ranged from 0.4 to 5.6 (mean 1.37 mg/dL). The postop BUN ranged from 11 to 120 (mean 36.44 mg/dL) and the creatinine ranged from 0.4 to 1.9 (mean 0.79 mg/dL). Follow-up ranged from 6 months to 6 years (mean 2.5 years). All patients were in good general condition at follow-up. Three had delayed developmental milestones and stunted growth. This was associated with raised BUN and creatinine levels.

Of the total 266 renal units, 31 were normal at presentation. Some patients had reflux or hydro-uretero-nephrosis (HUN) on one side with the other side being normal or had bilateral normal units. VUR was present in 57 patients (26 bilateral) (83 units). VUR was absent at repeat MCUG in 3 months–4 years (mean 1 year) in 41 units (49%). Of the remaining 42 units, 28 (34% of high grade VUR units) non- or poorly functioning kidneys were electively removed (*n* = 15) a year later or scheduled for removal (*n* = 13). Deflux injection was given in two patients and bilateral ureteric reimplantation was done in four others. Non-VUR slow draining obstructive/non-obstructive (HUN) was present in 108 patients (152 units), with 42 occurring bilaterally. Eleven renal units were poorly functioning. Post fulguration, slow but unobstructed drainage was seen in 23 (16%) units for a period of 1.5–5 years (mean 2.5 years); in the remaining it normalized by 3 months–2 years (13 months mean duration). Postoperatively, one patient underwent bilateral PCN followed by bilateral ureterostomy due to persistent raised BUN and poor general condition. He underwent unilateral nephrectomy and opposite side stoma closure 1.5 years later. Another neonate required vesicostomy in the immediate postoperative period for poor stream. A repeat MCUG and cystoscopy were normal 6 months later and the stoma was closed. The remaining patients had good urinary stream. Dribbling was present in seven patients and persisted for 6 months–3.5 years. Postop nocturnal enuresis was seen in four patients (3%) and they responded to oxybutinin/imipramine. There was a correlation between resolution of VUR, slow draining units and absence of urinary tract infections (UTIs) even if asymptomatic.

### Group B: Postop PU/BU ratio 2 SD–3 SD, range (1.626–1.92)

**Figure 2:**
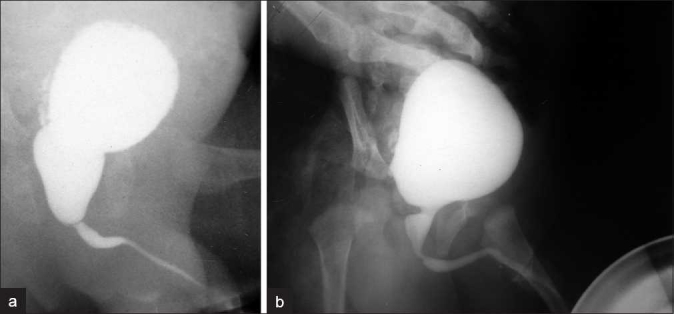
(a) Preop MCUG; (b) postop MCUG showing PU/BU ratio of 2–3 SD

Eleven patients (7%) had a postop PU/BU ranging from 1.67 to 1.86, with a mean of 1.734 [Figure 2a and b]. The preop ratio ranged from 2.5 to 30, with a mean of 12.295. Their age ranged from 2 months to 12 years (median 7 months; mean of 3.54 years). Six patients were below 1 year, with four less than 3 months age at presentation. The preoperative BUN ranged from 24 to 245 (mean 97.54 mg/dL) and the creatinine ranged from 0.5 to 3.9 (mean 1.636 mg/dL). The postoperative BUN ranged from 11 to 85 (mean 30.16 mg/dL) and the creatinine from 0.4 to 1.8 (mean 0.74 mg/dL).

Follow-up ranged from 6 months to 6 years, with an average of 2.5 years. All patients had a normal stream. Six children had no voiding dysfunction. One 10-year-old child had nocturnal enuresis for 1 year. Daytime frequency was seen in four children for 6 months–2 years (mean 1 year). Grade IV–V VUR was seen in five patients (three bilateral), which regressed completely by 3 months in five units. Of the remaining three persistent VUR units, two were seen in nonfunctioning units. In the non-VUR, HUN patients, slow (but unobstructed) drainage was persistent in two units (14%) at 3 years; in the remaining 12 units, they normalized by 1–2 years (mean 1 year 5 months). Asymptomatic UTI was seen in four patients for a period of 1–2 years. One patient had recurrent symptomatic UTI for 1.5 years. He had a severely trabeculated bladder with diverticulae on the 3-month postop MCUG also.

### Group C: Postop PU/BU ratio > 3 SD (>1.92)

**Figure 3:**
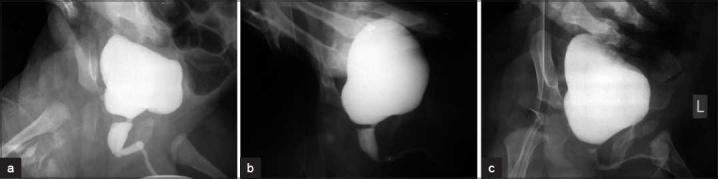
(a) Preop MCUG; (b) postop MCUG showing PU/BU ratio more than 3 SD post first fulguration; (c) post second fulguration MCUG showing less than 2 SD

Sixteen patients (10%) had a postop PU/BU ratio in the range 2–9.3, with a mean of 3.69 [Figure 3a–c]. Their preop ratio ranged from 3.5 to 32, with a mean of 10.45. Age ranged from 9 days to 8 years (mean 10 months). The preop BUN ranged from 15 to 240 (mean 55.4 mg/dL), while postoperative BUN ranged from 10 to 107 (mean 28.25 mg/dL). The preop creatinine ranged from 0.5 to 3.2 (mean 1.29 mg/dL) and postop creatinine ranged from 0.3 to 1.6 (mean 0.67 mg/dL).

Small severely trabeculated bladder was seen in 15 patients preoperatively. Postop severe trabeculation was seen in four patients while seven showed mild to moderate trabeculation. Preop, three units were normally functioning, six patients had VUR (three bilateral), and the remaining had slow draining units. After fulguration, VUR was still present in all the units at 6 months–3 year follow-up. Of these, two were poorly functioning units. Of the 20 units with HUN, only 3 (15%) had become normal at 1 year. The rest were persistent at 1–4 years (mean 2 years). Recurrent UTI was seen in five patients for 1–4 years.

Eight patients had retention (3), straining (5), poor stream (2), dribbling (6) and frequency (4). BUN/creatinine was above normal in four patients at follow-up. A second fulguration was performed in these patients within a period of 4–10 months, except in one patient who underwent a vesicostomy, a second fulguration being combined with vesicostomy closure 6 months later. These patients had a post-op PU/BU ratio between 2.2 and 9.3 (mean 4.51), the preop ratio being 4–20 (mean 8.63). After the second fulguration, the ratio reduced to a mean of 1.3 on follow-up MCUG. The urine stream as well as BUN/creatinine values, drainage of renal units on EC scan improved.

Three patients with a postop ratio of 2.33, 2 and 2.67 had actually developed a stricture and responded to urethral dilatation. Their postop ratio reduced to 1.25, 1.1 and 1.4, respectively. At 1 year follow-up, normal renal function and drainage was seen in two, while the other one had persistent unilateral HUN with grade V VUR.

In the remaining five patients, where no further procedure was done, postop ratio ranged from 2 to 5 (mean 3.22). The preop ratio had ranged from 8 to 32 (mean 17.2). Their age ranged from 11 days to 2 months (mean 1 month). These patients had a normal stream with no voiding dysfunction or UTI. There was associated dribbling only in one patient for 3 years. They have been followed up for 5 years now. Slow draining HUN persisted for 1–3 years.

Highly significant statistical differences [Table 2] are observed when the results are compared for the select parameters in the three groups: (1) Need for repeat fulguration – group A: 0/133 (0%); group B: 0/11 (0%); group C: 8/16 (50%). (2) Postop significant voiding problems – group A: 13/133 (9.8%); group B: 5/11 (45%); group C: 12/16 (75%). (3) Persistent HUN units – groups A and B: 25/168 (15%); group C: 17/20 (85%). (4) Persistent VUR not associated with poorly functioning kidney – group A: 14/42 (33%); group B: 1/3 (33%); group C: 7/9 (78%).

**Table 2:** Statistical significance of select parameters in groups A and B vs. group C

Parameter	Groups A and B	Group C	*P* value
Need for repeat fulguration	0 out of 144	8/16 (50%)	Highly significant
Postop significant voiding problems	18/144 (12.5%)	12/16 (75%)	<0.001 Highly significant
Persistent HUN (units)	25/168 (15%)	17/20 (85%)	<0.001 Highly significant
Persistent VUR (not associated with poorly functioning kidney	15/45 (33%)	7/9 (78%)	<0.01 Significant

### Group D: Analysis of referred patients after fulguration elsewhere

Twenty-one patients who had previously undergone intervention for PUV elsewhere were separately analyzed. Their age ranged from 2 months to 11 years (mean 27 months). Their preop BUN ranged from 18 to 278 (mean 65.4 mg/dL) and the creatinine from 0.2 to 3.4 (mean 1.38 mg/dL). After management at our center, the BUN was 15–86 (mean 38.5 mg/dL) and the creatinine was 0.2–1.8 (mean 0.84 mg/dL). The preop PU/BU ratio ranged from 2 to 22 (mean 7.92) and the postop ratio from 0.25 to 5 (mean 1.82).

These patients presented with recurrent febrile UTI (5), poor stream (3), retention (2), incontinence (1), frequency (1), hematuria (1), flank pain (1). Apart from fulguration, these patients had undergone multiple procedures, namely, vesicostomy (6), ureterostomy (4), ureteric reimplantation (2), suprapubic cystostomy (2), one of which was after extravasation of urine after transurethral fulguration (TUF), PCN (three units), vesicostomy closure (2), removal of bladder calculus (1), ureterostomy closure followed by local leakage of urine (1). Two patients had undergone fulguration twice. Two patients had developed urethral stricture, one of which was probably secondary to prolonged catheterization. Another had a urethral fistula, post TUF.

Nineteen patients underwent repeat fulguration (at our center) along with urethral fistula closure (1), urethral dilatation (2), and ureterostomy closure (1). One ureterostomy closure site leak closed spontaneously after redo fulguration. One child who had no residual valves continued to have incontinence and was started on oxybutinin without much relief. The stream improved in all patients. One patient had asymptomatic UTI for 6 months; in the rest there were no infections. Three patients had nocturnal enuresis for 1–2 years. Before admission, 11 units had slow draining HUN, 22 had VUR. 3 months–1 year after the second fulguration, the HUN was not seen at follow-up in six units and showed improved drainage pattern in the others. Among VUR patients, in eight units the reflux had subsided. The remaining underwent nephroureterectomy (3), bilateral ureteric reimplantation (1) and bilateral deflux injection (1). Three are persisting in poorly functioning kidneys.

## DISCUSSION

Early endoscopic valve fulguration is the best initial modality of management in the majority of children with PUVs and this gives superior results to diversion procedures.[1] Dysplasia of renal units, preexisting VUR and its effect on renal parenchyma are irreversible and not amenable to the surgical skill. Very contracted/fibrotic urinary bladders also are difficult problems. Adequacy of fulguration vis-à-vis the goal of achieving a good urinary stream and avoiding valve bladder syndrome has not been addressed in the literature.

After fulguration, many surgeons are satisfied by asking the parents about the urinary stream. Although this is an important tool, it could be highly subjective. On a comparative basis, it would be definitely better than the preoperative status. This is especially true in small babies and infants who are still in diapers. Although not universally done, repeat MCUG is expected to be performed 3 months after valve ablation to exclude the possibility of post-fulguration stricture, remnant valves and reassess VUR.[2,3]

As per the literature, dilatation of the PU is generally expected to persist for considerable periods of time. In a recent paper, Smeulders *et al*. have given a positive predictive value of a repeat MCUG for subsequent resection of valve remnants to be only 56%,[4] in a study of 31 patients. However, their data of posterior urethral dilatation on repeat MCUG showed that when there was persistent dilatation, repeat valve ablation had to be done in 8 out of 12 cases (67%). When the dilatation was reduced, repeat valve fulguration was necessary in 4 out of 8 cases (50%), but when dilatation was resolved, repeat valve ablation was necessary in only 4 out of 10 cases (40%). This indicates that there is a direct correlation of persistent PU dilatation and inadequate fulguration.

Our previous experience[5] had however shown that along with the rapid improvement in the clinical status of the child, there is a significant reduction in the dimensions of the PU and an increase in the dilatation of the anterior urethra, especially the bulbar urethra, after a successful valve fulguration. As the pre- and post-fulguration MCUG may be taken by different X-ray machines and in a different setup, a ratio was developed in 2003–2005 wherein two parameters in the same study would be compared before surgery and 3 months after fulguration, i.e., the ratio of the diameter of PU to that of the BU. This ratio is a useful tool in predicting severity of the disease and objectively assesses the adequacy of fulguration independent of the surgeon’s opinion.[5,6]

Bani Hani had done a similar study in 20 children with PUV.[7] The postoperative ratio given by them compatible with adequate fulguration was 3.1 (range 1.9–4). No comparison was done with the preoperative ratio. Gupta *et al*., in a study of 30 patients, have also given a post-fulguration ratio of 2.5–3 as an acceptable result.[8] The effect of the fulguration on the voiding dysfunction and drainage of renal units has not been adequately studied in these reports. Moreover, these ratios as per our study are too high and not compatible with adequate decompression. Most children with PU/BU ratio >1.92 had significant residual problems.

### Vesicoureteral reflux

In our study, VUR was present in 68 of the 160 patients [32 bilateral patients (47%) and 36 unilateral; total 100 units]. After fulguration, in 49.4% patients in group A and 62.5% patients in group B, VUR had completely regressed. In an earlier study also, we had demonstrated that 31.5% VUR subsided within 3 months of fulguration and 79% by 6 months.[9] Two-thirds of persisting VUR in groups A and B were in poorly functioning units. There is a strong association between adequate fulguration and regression of VUR as well as between persistence of VUR and poorly functioning units.

The patients who had a PU/BU ratio <2 SD of the normal had a very low incidence of persistent HUN compared to those with >2 SD of normal. This was also true for postop voiding dysfunction and metabolic problems in spite of the initial serious nature of disease. There is an interesting observation. A small group of 10 neonates and infants (17 days–11 months with a mean 3.5 months) (5 each from groups B and C) with a high preop PU/BU ratio ranging from 11 to 32 (mean 18.57) had good results with respect to normal stream, significant reduction of HUN within 1 year postop, and no voiding dysfunction in spite of a postop PU/BU ratio >2 SD (1.71–5; mean 2.62). In these patients, the preop ratio was 5.24–11.97 (mean 7.74) times the postop ratio. If there is a very high preop posterior urethral dilatation, gross reduction in size (not necessarily reaching normal levels) is associated with good prognosis. However, we would like to point out that 62% of group A patients were neonates and infants with a pre-op PU/BU ratio ranging from 2-32 (mean 7.89) which was 1.9-35 times (mean 10.74) of the post-op ratio. Inspite of this, their PU/BU ratios normalized after adequate fulguration.

Much has been written and discussed about the valve bladder syndrome associated with a noncompliant bladder wall and acquired nephrogenic diabetes insipidus causing increased urine output, which secondarily leads to HUN and incontinence.[10] Koff *et al*. once accepted persistent dilatation as residual upper tract stretching but now do not.[11] Of the patients referred from other centers after fulguration, the sequelae of so-called valve bladder syndrome was seen often. These patients not surprisingly had a high PU/BU ratio of 2–22 (mean 7.92). A case in point is a 7–year-old boy fulgurated twice by another surgeon, who presented with flank pain and urinary retention. After fulguration done by us, the stream improved but the child still had persistent nocturnal enuresis and bilateral HUN with slow drainage. He was showing all the features of a valve bladder syndrome. Perusal of previous MCUGs showed inadequate fulguration and this status persisted for a very long period of time, 6 years in this case. This child’s urea/creatinine has come down from previous levels but he continues to have UTI off and on when the BUN/creatinine levels rise temporarily. Symptomatic UTIs appear to have a direct correlation with persistent slow draining systems and as these improve or become normal, the incidence of UTI also reduces. The complication of UTI leading to uremia would be much higher in patients with a single functioning but slowly draining system.

We do not subscribe to the view that “bladder neck hypertrophy” is a cause of poor results, even after adequate fulguration. To substantiate this point, in Figure 3(a), there is an apparent appeareance of a significant bladder neck hypertrophy which persisted in Figure 3(b) i.e. after an inadequate valve fulguration. However, this disappeared in Figure 3(c) after adequate fulguration.

Under fulguration rather than over fulguration is always stressed in the literature. While it is true that over zealous fulguration or fulguration by an inexperienced surgeon can create urethral stricture or fistula, we have repeatedly found that patients who have complete fulguration in the first sitting invariably do better than those who have inadequate fulgurations.

Our experience has been that a period of catheterization often induces inflammation leading to bleeding and reduced vision at the time of fulguration which can jeopardize the adequacy of fulguration. Preop catheterization should therefore be avoided unless strongly indicated. A method we also follow to immediately check the completeness of fulguration is to demonstrate the stream on table by suprapubic compression after fulguration. Adequate fulguration should improve the stream to near normal. This is followed 3 months later by performing a MCUG in the operation theater under aseptic precautions and general anesthesia. This markedly reduces the discomfort and secondary UTI associated with a MCUG in the radiology department.

In this prospective study, initially, patients were re-scoped depending on the symptoms (poor stream and recurrent UTI) only. The significance of the ratio became more apparent as the study progressed. In this series, a second fulguration was performed in a total of 8 patients (5%) previously fulgurated by us. Imaji *et al*. gave an incidence of 47.4% of second fulguration in patients who had a severe obstructing membrane.[12] In the data presented, one can see a pattern of good clinical result if one can do adequate fulguration and achieve normalization of PU. Conversely, if the fulguration is inadequate, PU/BU ratio remains high and the problems with voiding continue. A strong relationship exists between inadequate fulguration and slow draining HUN, urosepsis and abnormal renal function. Therefore, it is of paramount interest to assess the completeness of the fulguration by a properly done MCUG as early as possible after fulguration, as delay might lead to an irretrievable situation. A simple calculation of the PU/BU ratio would be a good guide for adequacy of fulguration and to take the patient up for a second look cystoscopy in a symptomatic patient.
